# The causal relationship between osteoarthritis and bladder cancer: A Mendelian randomization study

**DOI:** 10.1002/cam4.6829

**Published:** 2023-12-15

**Authors:** Xi Zhang, Zengjin Wen, Zixuan Xing, Xiaoyu Zhou, Zhiluo Yang, Ruijun Dong, Jiao Yang

**Affiliations:** ^1^ Department of Medical Oncology The First Affiliated Hospital of Xi'an Jiaotong University Xi'an China; ^2^ School of Basic Medical Sciences, Qingdao Medical College Qingdao University Qingdao China; ^3^ Department of Infectious Diseases The Second Affiliated Hospital of Xi'an Jiaotong University Xi'an China

**Keywords:** any osteoarthritis, bladder cancer, hip osteoarthritis, knee osteoarthritis, Mendelian randomization

## Abstract

**Objective:**

The causal association between osteoarthritis (OA) and bladder cancer remains unclear. This Mendelian randomization (MR) study was carried out to assess the potential causal effects of any OA, knee OA and hip OA, and bladder cancer.

**Method:**

Genome‐wide association study (GWAS) summary data for OA and bladder cancer were obtained in GWAS CATALOG, UK Biobank, and FinnGen Consortium. Inverse‐variance weighted (IVW) approach was primarily conducted to evaluate the causal relationships between OA and bladder cancer, and MR‐Egger intercept and Cochran's *Q* test were mainly used to estimate heterogeneity and pleiotropy. MR‐PRESSO was used to test the presence of horizontal outliers. Leave‐one‐out analysis was utilized to ensure the reliability of the results.

**Results:**

A higher genetic predisposition to any OA has a causal association with bladder cancer risk, while neither knee OA nor hip OA is causally linked to bladder cancer. MR‐Egger intercept analysis exhibited that any OA and knee OA had no pleiotropic effect on the risk of bladder cancer, and Cochran's *Q* test showed that any OA, knee OA and hip OA had no heterogeneity on bladder cancer risk. Neither MR PRESSO analysis nor leave‐one‐out analysis revealed any outlier SNPs.

**Conclusions:**

This MR study exhibited a positive cause‐and‐effect relationship between any type of OA and bladder cancer risk, but not between site‐specific OA, knee OA and hip OA, and bladder cancer. Attention should be paid to the screening and prevention of bladder cancer in OA patients at any site.

## Abstract 2

The causal association between osteoarthritis (OA) and bladder cancer remains unclear. This Mendelian randomization (MR) study was carried out to assess the potential causal effects of any OA, knee OA and hip OA, and bladder cancer.

## 1 INTRODUCTION

Osteoarthritis (OA), as one of the most prevalent degenerative joint diseases worldwide, represents a significant cause of disability and increased socioeconomic costs in the elderly.^1^, ^2^ Composed of various metabolic and inflammatory factors, OA is clinically characteristic of swelling, morning stiffness, chronic pain, loss of mobility, and radiological findings in diarthrodial joints, especially the knee and hip.^3^, ^4^, ^5^ In recent years, the causal relationship between OA and cardiovascular diseases,^6^, ^7^, ^8^ cardiovascular risk factors,^9^, ^10^, ^11^, ^12^, ^13^ stroke,^14^ senile central nerve system dysfunction,^15^ and gout^16^ has attracted more and more attention. Additionally, the relationship between OA and cancer remains to be studied and elaborated, as both exhibit immune response, chronic pain, and disorder later in life, with longer courses of treatment and a greater health burden.^17^, ^18^

Bladder cancer is one of the top 10 most common cancers, with an estimated 84,825, 91,893 and 204,000 new cases in the United States and China during 2022 and in Europe during 2020.^19^, ^20^ Renowned as the most expensive malignancy, bladder cancer becomes a significant healthcare problem that often requires costly lifelong surveillance and repeated treatment due to its high reoccurrence rate and little treatment progress.^21^, ^22^ Well‐documented risk factors for bladder cancer include smoking and environmental and occupational exposure to specific chemicals, especially carcinogens.^23^, ^24^ A population‐based retrospective cohort study indicated that individuals with knee or hip osteoarthritis (KHOA) had higher risks of bladder cancer. However, the study also mentioned that the effect of KHOA on the increased risk of bladder cancer was probably mediated through nonsteroidal anti‐inflammatory drugs (NSAIDs).^25^ In addition, a large real‐world matched cohort study reported that knee and hip OA were not associated with bladder cancer.^26^ The actual causal relationship between OA and bladder cancer remains unclear. Therefore, it is essential to probe into the connection between OA and bladder cancer, so as to prevent the appearance and development of bladder cancer and improve the high burden and mortality of bladder cancer. Unsatisfactorily, it is rarely feasible and unethical to conduct randomized controlled trials (RCTs) to assess the effect of OA on bladder cancer risk.

Herein, Mendelian randomization (MR) is an emerging and valid genetic epidemiological design that enhances causal inference and overcomes bias from residual and unknown confounding, reverse causation and measurement error by applying genetic variants, which are randomly assigned single nucleotide polymorphism sites (SNPs) at conception, as instrumental variables (IVs) for exposure (e.g., OA) on an outcome (e.g., bladder cancer).^27^, ^28^, ^29^ The selected SNPs must satisfy three key assumptions: (1) relevance, they are strongly associated with the exposure such as any OA, knee OA and hip OA; (2) exclusion restriction, they are only associated with the outcome (bladder cancer) through the OA exposure; and (3) independence, they are not associated with confounders of the exposure (OA)‐outcome (bladder cancer). Furthermore, based on the detailed phenotypes provided by large‐scale genome‐wide association studies (GWAS), MR allows greater exploration of genetic susceptibility to bladder cancer or OA at any site or site‐specific OA.

As far as we know, this is the first two‐sample univariate MR study to investigate the causal effects of genetic liability for any site OA, site‐specific OA (knee OA and hip OA) on bladder cancer risk.

## 2 MATERIALS AND METHODS

### 2.1 Study overview

**FIGURE 1:**
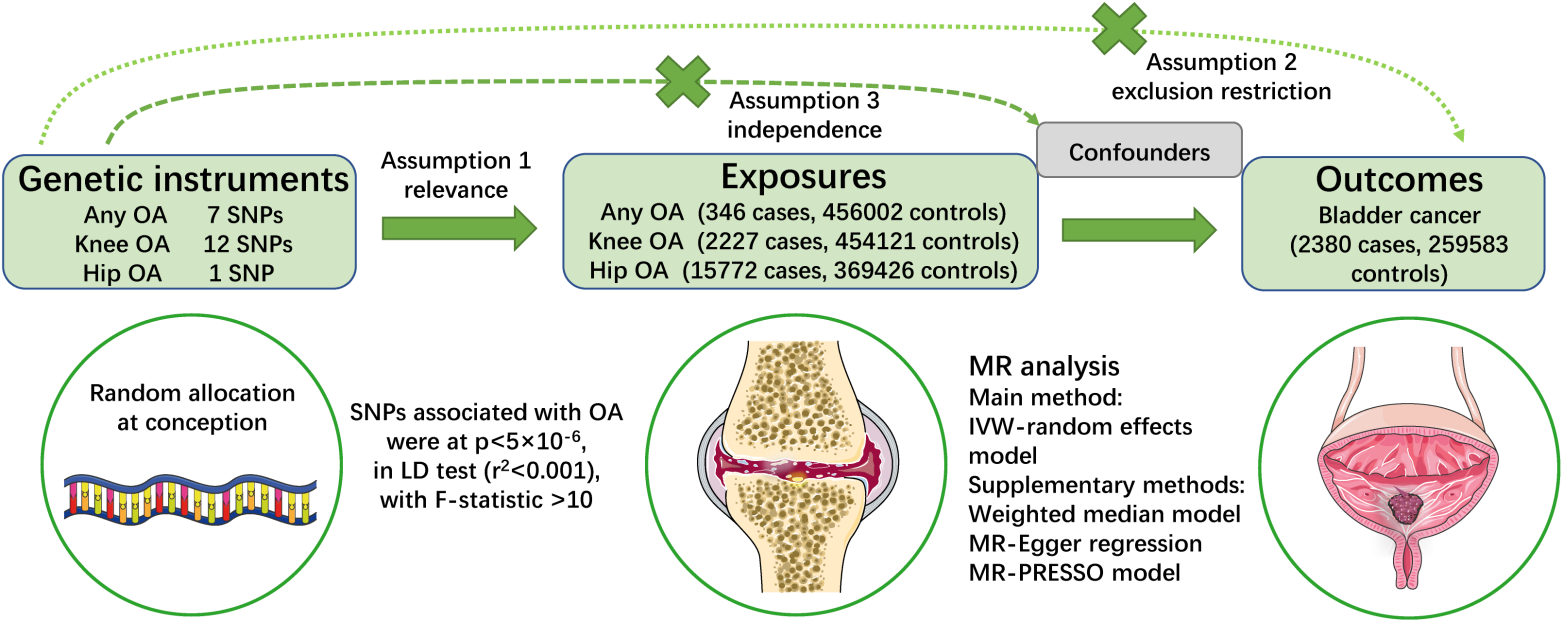
The design overview of MR study. SNPs selected as instrumental variables must fulfill the three assumptions: (1) relevance, they are robustly associated with the exposure such as any OA, knee OA and hip OA; (2) exclusion restriction, they are only associated with the outcome (bladder cancer) through the OA exposure; and (3) independence, they are not associated with confounders of the exposure (OA) and outcome (bladder cancer). IVW, inverse‐variance weighted; LD, linkage disequilibrium; MR, Mendelian randomization; OA, osteoarthritis; SNP, single‐nucleotide polymorphism.

A MR was conducted to explore the potential causal effects of OA on bladder cancer. Figure 1 shows an overview of the study design. Since the research data were obtained from public databases, extra ethical approval was not required.

### 2.2 Data resources and selection of SNP for OA

The tabulated statistical data of OA at any site and knee OA was derived from a published study of GWAS CATALOG involving a total of 456,348 European‐decent individuals,^30^ and a publicly available data of hip OA was acquired from another genome‐wide study conducted by the UK Biobank including 385,198 European‐decent individuals.^31^ We firstly extracted the SNPs that reached genome‐wide significance (*p* < 5 × 10^−8^) for any OA, knee OA and hip OA, but because of a limited number, we broadened the threshold to 5 × 10^−6^ to extract. Meanwhile, SNPs were checked for independence by using linkage disequilibrium (LD) test (*r* ^2^ < 0.001). In addition, *F*‐statistics and *R* ^2^ for each SNP were calculated, and the formula of *F*‐statistics is = *R* ^2^(*N* − 2)/(1 − *R* ^2^).^32^ SNPs would be eliminated from MR analysis if their *F*‐statistics were less than 10. Then, we analyzed the potential confounding factors associated with the selected SNPs by searching the PhenoScanner V2 web site (http://www.phenoscanner.medschl.cam.ac.uk/).

### 2.3 Data source for bladder cancer

We obtained the aggregate data for bladder cancer from the FinnGen Consortium including 2380 cases and 259,583 controls with a median age of 69 years. The FinnGen Consortium aims to construct a resource which can provide new insights into disease genetics.^32^ Logistic regression was used to calculate genetic association estimates.^33^

### 2.4 Statistical analysis

In this study, we adopt the two‐sample MR analysis to study the causal associations between OA and bladder cancer. Inverse‐variance weighted (IVW) approach was used for the principal analysis, which calculates the Wald ratio and conducts a meta‐analysis for the combined casual effect by using the inverse‐variance of SNPs as weights.^34^ Since hip OA had only one SNP in this analysis, Wald ratio was applied to substitute for IVW approach. In addition, we used other MR methods for sensitivity analysis to make a robust result. Among the methods, weighted median can combine data on numerous genetic variants into a single causal estimate, which is concordant even when as much as 50% of the information comes from noneffective IVs.^35^ Besides, MR Egger regression was adopted to test for bias from pleiotropy, which can still give an effective test of the null causal hypothesis and a consistent causal effect estimate even though all the genetic variants are invalid IVs.^36^ If the *p*‐value of the egger intercept is greater than 0.05, no potential pleiotropic effects is suggested. MR‐PRESSO approach was adopted to test the existence of horizontal outliers. Cochran's *Q* test was adopted to quantify the level of heterogeneity of each SNPs. If *p*‐value <0.05, there was heterogeneity between the genetic instruments. Leave‐one‐out analysis was utilized to guarantee the reliability of the results by removing each SNP at turn and estimating the integrated effects of the remaining SNPs.

Given the problem of multiple testing, the threshold for Bonferroni‐corrected *p*‐value was 0.017 (0.05/3). We adopted R (Version 4.2.2) software and the “TwoSample MR” package for statistical analysis.

## 3 RESULTS

First, we obtained 7, 14 and 2 LD‐independent (*r* ^2^ < 0.001) IVs that achieved significant threshold (*p* < 5 × 10^−6^) from any OA, knee OA and hip OA, respectively. All of the *F*‐statistics for IVs were larger than 10, meaning that selected IVs were powerful enough. Besides, the selected IVs were not significantly associated with bladder cancer (*p* > 5 × 10^−6^), indicating that IVs cannot be directly involved in bladder cancer (Tables S1–S3). After searching the PhenoScanner V2 web site, two SNPs (rs111623565, rs11655443) for knee OA and one SNP (rs1800562) for hip OA were excluded (Tables S4–S6). Finally, 7 SNPs, 12 SNPs, and 1 SNP were made available for OA on any site, knee OA and hip OA, respectively. Detailed information on the relationship between the selected SNPs and exposures is shown in Tables 1, 2, 3.

**TABLE 1:** Characteristics of SNPs for any OA from GWAS meta‐analysis.

SNP	Chr	Pos	Closest gene	EA	OA	EAF	*β*	SE	*p*‐value
rs10042496	5	180423816	GFPT2	C	T	0.119	0.588	8.152	3.85E‐06
rs150519705	14	34956419	SRP54	A	G	0.009	2.330	2.506	1.74E‐06
rs4328897	4	154093204	DCHS2	C	A	0.468	0.371	12.977	1.69E‐06
rs647917	2	8412198	ID2	C	T	0.322	0.396	11.923	2.83E‐06
rs77491146	3	65414440	MAGI1	C	T	0.078	0.701	7.005	3.17E‐06
rs79756454	10	2245630	ADARB2	C	T	0.033	1.188	4.643	7.54E‐07
rs9287903	2	168823612	NOSTRIN	C	T	0.262	0.458	11.539	2.51E‐07

Abbreviations: Chr, chromosome; EA, effect allele; EAF, effect allele frequency; OA, other allele; Pos, position; *p*‐value, *p*‐value for the genetic association; SE, standard error.

**TABLE 2:** Characteristics of SNPs for knee OA from GWAS meta‐analysis.

SNP	Chr	Pos	Closest gene	EA	OA	EAF	*β*	SE	*p*‐value
rs111956618	12	94985715	NDUFA12	C	A	0.126	−0.229	21.912	6.44E‐07
rs12792833	11	58777845	GLYAT	T	G	0.116	0.217	21.246	4.48E‐06
rs140144990	3	9610618	MTMR14	G	T	0.011	0.687	7.063	4.48E‐06
rs143339839	2	33221366	LTBP1	G	A	0.039	0.410	12.948	2.59E‐07
rs148504141	12	51365679	GALNT6	C	G	0.009	0.776	6.294	4.78E‐06
rs16944492	18	435867	COLEC12	C	T	0.039	0.394	12.743	9.65E‐07
rs4696079	4	151342678	SH3D19	A	C	0.463	−0.145	33.072	1.72E‐06
rs55642448	8	10607245	RP1L1	T	C	0.387	0.143	32.342	3.64E‐06
rs56103030	10	29311938	LYZL1	A	G	0.018	0.563	8.801	2.04E‐06
rs71604079	4	37577483	C4orf19	A	G	0.016	0.574	8.409	3.77E‐06
rs740046	7	31480533	CCDC129	G	A	0.366	−0.160	31.291	6.17E‐07
rs7784284	7	127515134	GCC1	A	G	0.153	0.194	23.912	3.75E‐06

Abbreviations: Chr, chromosome; EA, effect allele; EAF, effect allele frequency; OA, other allele; Pos, position; *p*‐value, *p*‐value for the genetic association; SE, standard error.

**TABLE 3:** Characteristics of SNPs for hip OA from GWAS meta‐analysis.

SNP	Chr	Pos	Closest gene	EA	OA	EAF	*β*	SE	*p*‐value
rs17610181	17	61590592	NACA2	A	G	0.150	0.099	0.016	6.59E‐10

Abbreviations: Chr, chromosome; EA, effect allele; EAF, effect allele frequency; OA, other allele; Pos, position; *p*‐value, *p*‐value for the genetic association; SE, standard error.

### 3.1 Causal association between OA and bladder cancer

**FIGURE 2:**
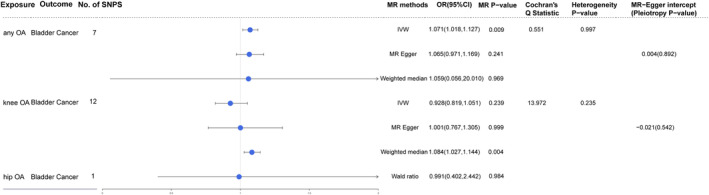
MR estimates from each method of assessing the causal effects of any OA, knee OA and hip OA on bladder cancer. CI, confidence interval; IVW, inverse‐variance weighted; MR, Mendelian randomization; OA, osteoarthritis; OR, odds ratios; SE, standard error.

**FIGURE 3:**
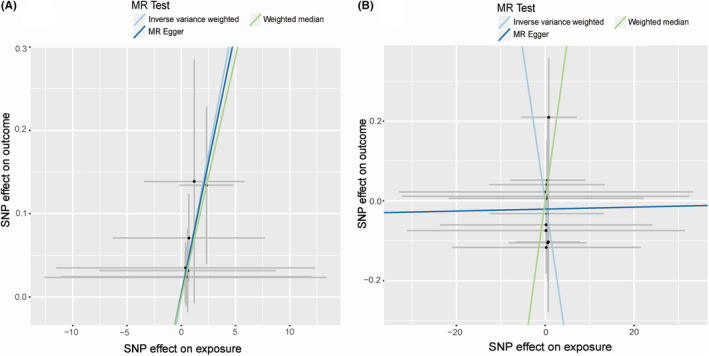
Scatter plot of the causal associations of any OA and knee OA with bladder cancer using different MR methods. Scatter plot showing the effect sizes (beta) of the SNP effects on outcomes (*y*‐axes) and the exposure (*x*‐axes) with 95% confidence intervals. Causal effects for (A) any OA on bladder cancer and (B) knee OA on bladder cancer. MR, Mendelian randomization; OA, osteoarthritis.

As shown in Figure 2, there was a significant association between any OA and bladder cancer (OR: 1.071, 95% CI: 1.018–1.127, *p* = 0.009) by using the IVW method. Although the *p*‐value of MR Egger and weighted median method did not reach the significant threshold, OR showed a consistent result that any OA can increase the risk of bladder cancer (MR Egger OR: 1.065, 95% CI: 0.971–1.169; weighted median OR: 1.059, 95% CI: 0.056–20.010). The scatter plot for effect sizes of SNPs for any OA is shown in Figure 3A. The OR values showed that knee OA and hip OA were negatively correlated with bladder cancer (OR of knee OA: 0.928, 95% CI: 0.819–1.051; OR of hip OA: 0.991, 95% CI: 0.402–2.442). However, the result was not statistically significant (*p*‐value of knee OA: 0.239; *p*‐value of hip OA: 0.984). In other words, there was no causal relationship between knee OA, hip OA, and bladder cancer. The scatter plot for effect sizes of SNPs for knee OA is shown in Figure 3B. The scatter plot of the hip OA cannot be made due to the limited number of SNPs.

### 3.2 Sensitivity analysis

**FIGURE 4:**
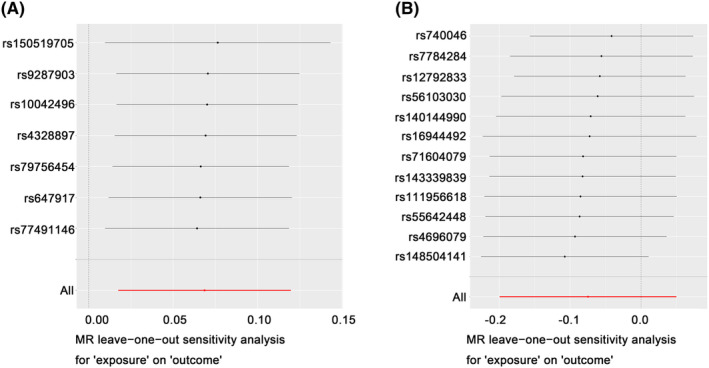
The leave‐one‐out analysis plots of the causal associations of any OA and knee OA with bladder cancer. This figure shows the Mendelian randomization estimated effect sizes for (A) any OA and (B) knee OA, presenting the casual relationship between any OA and bladder cancer was independent of a single SNP. OA, osteoarthritis.

Hip OA was excluded from sensitivity analysis since the SNPs were <3. In accordance with Cochran's *Q* test (Figure 2), there was no heterogeneity in any OA (*p* = 0.997) and knee OA (*p* = 0.235). In the MR Egger analysis, the evidence of horizontal pleiotropy was not observed in any OA (*p* = 0.892) and knee OA (*p* = 0.542). No outlier can be observed in MR PRESSO analysis for any OA (*p* = 0.9996) and knee OA (*p* = 0.374). What's more, the result of leave‐one‐out analysis showed that the casual effect between any OA and bladder cancer was not related to single SNP. The leave‐one‐out analysis plots for any OA and knee OA are shown in Figure 4.

## 4 DISCUSSION

This is the first large population‐based MR analysis to determine a causal effect between any OA, knee OA and hip OA with bladder cancer risk. Our findings reveal that the predisposition toward any OA is causally associated with a higher risk of bladder cancer, indicating OA might promote the occurrence and development of bladder cancer. However, site‐specific OA, particularly knee OA and hip OA, did not have a causal effect on bladder cancer risk. More modestly, we suggest that the causal association between OA and bladder cancer is a total effect rather than a direct and specific effect, where the mechanical pathways might be complex and unclear. Therefore, the biological implication of any OA on bladder cancer risk should be illustrated, and individual screening and prevention strategies for bladder cancer in patients with OA should be adopted.

Numerous studies have examined the link between OA and several types of cancer, including lung, colon, and prostate cancer. However, the results are different and controversial.^25^, ^26^, ^37^, ^38^, ^39^, ^40^ Specifically, the current MR analysis is completely inconsistent with observational evidence for OA and bladder cancer risk.^25^, ^26^ In a large‐scale matching cohort study, patients with an increased duration of knee OA had an elevated risk of bladder cancer with a hazard ratio (HR) close to one, and hip OA was not associated with bladder cancer.^26^ In addition, in a national retrospective cohort study, there was a higher risk of bladder cancer in patients with knee and hip OA.^25^ Research on surgical treatment after OA and bladder cancer risk is incomplete.^41^, ^42^ Back in 1996, among 15 follow‐up patients who underwent atlantoaxial or occipitocervical arthrodesis, one in two deaths was due to complications from bladder cancer, suggesting arthrodesis after OA might increase bladder cancer mortality.^41^ Differently, there was no increase in the overall risk of bladder cancer after total hip arthroplasty in a large and well‐defined group of people followed up over a long period of time.^42^ The different findings relative to this MR study may be due to shortcomings in traditional observational methods, especially the residual and unknown confounding. In addition, the specific position and degree of OA are often inaccurate, recall bias is inevitable, and the cost of data collection and analysis is high in observational studies. Moreover, the existing literature has not demonstrated overall associations between any OA and bladder cancer. And RCTs are ideal but impractical in terms of exploring causality because they require plenty of time, money, human, and material resources.

Indeed, it is more practical to collect evidence through the MR approach to fully illustrate the causal effects of genetic IVs for any OA, knee OA and hip OA on bladder cancer risk. Compared with retrospective and matched cohort studies, the MR analysis can mitigate the influence of reverse causal association, social environment, and lifestyle factors. Moreover, 7 SNPs, 12 SNPs, and 1 SNP were selected as the IVs in OA on any site, knee, and hip under three assumptions. The accuracy and reliability of MR analysis and sensitivity analysis can be improved by applying different algorithms in sequence. In MR analysis, a significant causal association between any OA and bladder cancer risk was supported by the IVW result (OR: 1.071, 95% CI: 1.018–1.127, *p* = 0.009), but not by the weighted median (OR: 1.059, 95% CI: 0.056–20.010, *p* = 0.969) methods and MR‐Egger method (OR: 1.065, 95% CI: 0.971–1.169, *p* = 0.241), because the IVW analysis had higher priority and more robust intercept and slope than the MR‐Egger and weighted median. Meanwhile, there were no significant effects between knee OA (IVW OR: 0.928, 95% CI: 0.819–1.051, *p* = 0.239; MR Egger OR: 1.001, 95% CI: 0.767–1.305, *p* = 0.999; weighted median OR: 1.084, 95% Cl: 1.027–1.144, *p* = 0.004) and hip OA (IVW OR: 0.991, 95% CI: 0.402–2.442, *p* = 0.984) and bladder cancer. Furthermore, MR‐Egger intercept analysis exhibited that any OA (*p* = 0.892) and knee OA (*p* = 0.542) had no pleiotropic effect on the risk of bladder cancer, and Cochran's *Q* test showed that any OA (*p* = 0.997) and knee OA (*p* = 0.235), had no heterogeneity on bladder cancer risk. Based on comprehensive and thorough tests, the overall conclusions of our study were unlikely to be strongly influenced by bias.

Due to the scarcity of epidemiological and laboratory studies on OA and bladder cancer, the underlying mechanism of the causal association between any OA and bladder cancer remains complicated and confusing. However, several factors may explain the findings. First, inflammatory response plays an indispensable role in the pathogenesis of both OA and bladder cancer.^43^, ^44^ During OA, various pro‐inflammatory cytokines such as interleukin (IL)‐1β/15/17, tumor necrosis factor (TNF)‐α, nitric oxide (NO), and matrix metalloproteinases (MMPs) are elevated, which indicate local and systemic inflammatory responses and promote articular cartilage destruction and tissue remodeling.^45^ And a chronically inflammatory environment contributes to neoplastic transformation and the progression of bladder cancer.^46^ In the interpretation of causal association between any OA and bladder cancer, OA at any site may imply a wider spread of inflammatory responses and higher expression of inflammatory cytokines, which are risk predictors and drivers of bladder cancer. Second, genetic variants, particularly SNPs of any OA, were used as IVs in this MR analysis, referring that genetic susceptibility to any OA and systematic inflammatory predisposition might be risk factors for bladder cancer at the genetic level. Third, some drugs excreted through the urinary system during the diagnosis and treatment of OA may become carcinogens of bladder cancer.^25^, ^47^, ^48^, ^49^ In addition, a number of other risk factors are shared in the development of OA and bladder cancer, such as obesity and metabolic syndrome,^50^, ^51^, ^52^, ^53^ coffee consumption,^33^, ^54^ higher serum IGF‐1 concentration,^55^, ^56^, ^57^ Brazilin,^58^ Chondromodulin‐1,^59^ vitamin K‐dependent protein, and GRP/Ucma.^60^

The mechanism explanation for the noncausal association between site‐specific OA (knee OA and hip OA) and bladder cancer risk may be related to the higher dose or longer duration of NSAIDs. Case–control, prospective and retrospective studies demonstrated significant association between non‐aspirin NSAIDs and reduced risk of bladder cancer, indicating the preventive and protective effects of NSAIDs, especially ibuprofen, acetaminophen, naproxen, NO‐naproxen, and sulindac on bladder cancer.^61^, ^62^, ^63^, ^64^, ^65^ As the specific and susceptible parts of OA, knee and hip OA easily present uncomfortable and painful sensation and chronical movement disorders, which are more likely to attract people's certain attention and thus lead to the use of chronic NSAIDs.^66^, ^67^, ^68^ The attenuated effect of chronic use of NSAIDs in knee and hip OA on bladder cancer risk may counter the increased risk of inflammatory response for bladder cancer, thus showing no causal association overall. Relative to site‐specific OA, any site OA might present more hidden, unappreciated, delayed‐treated and widespread inflammation that was not fully confronted by NSAIDs, thereby possibly manifesting a true effect—causal effect of OA on bladder cancer risk.

Because OA and bladder cancer are widespread diseases with significant health and economic burdens, it is critical to look for a link between them from a public health perspective. In the general middle‐aged population, early screening for OA status and traditional bladder cancer risk factors can be considered for early diagnosis and intervention to reduce future bladder cancer events. Among clinicians, bladder cancer risk should be considered to some extent when treating and following up for OA patients. At the same time, in patients with any OA, attention should be paid to the risk of bladder cancer and other bladder cancer risk factors should be kept away. Epidemiologists and laboratory researchers may need to attach some importance to the causal association and underlying mechanism of OA and bladder cancer. To further investigate the relationship between OA and bladder cancer, the anticancer research on OA drugs, especially NSAIDs, may offer new understanding about the prevention and treatment of bladder cancer. In fact, reducing the incidence of OA directly may not be a meaningful clinical practice for decreasing the risk of bladder cancer, as some methodological researchers did not recommend interpreting the causal effect by MR approach as the intended effect of intervention risk factors in a clinical setting, or even proposing estimates of causal effect at all.^69^, ^70^, ^71^

Several limitations and improvements should be taken into account in our study. First, MR instrumentation of OA remains challenging because of exposure phenotypic complexity and uncertainty about the biological function of genetic variants. We only used any OA, knee OA and hip OA as the three types of exposure phenotype in this MR study; further stratification of exposure phenotypes (e.g., self‐reported and clinically confirmed OA, newly diagnosed OA and chronic treated OA, OA at individual sites) with bladder cancer risk remains to be explored. Second, it is difficult and not feasible to conduct MR analysis based on different age, sex, height, stratum, and ancestry on account of the limitations and deficiencies of the GWAS summary statistics. Only participants of European ancestry were included in this study (especially those from the United Kingdom and Finland), and IVs found in European ethnicity cannot be transferred to non‐European ethnicity, so further MR studies of other population are needed to elaborate the causality. Third, the extraction criteria for SNPs with genome‐wide significance are relatively loose (*p* < 5 × 10^−6^ far more than 5 × 10^−8^), the results of MR Egger and weighted median were not consistent with the IVW method in MR analysis of any OA on bladder cancer. Under strict inclusion criteria, larger sample GWAS are required to obtain more accurate SNPs, weighted median might also support IVW's causal result. Fourth, it is unclear whether the treatment of OA, specifically prolonged NSAID consumption and joint replacement, impacts the link between OA and the possibility of developing bladder cancer. Fifth, due to the limitations of summary‐level data and the research methods, no model adjustment was made to explain whether the causal association between OA and bladder cancer has a linear or nonlinear relationship. Sixth, for OA at specific sites, we only explored the causal association between the more important knee OA and hip OA and bladder cancer, without further exploring other types, which reduces the clinical relevance of the relationship between OA at specific sites and bladder cancer. However, some cohort studies based on nationwide population have indicated that ankylosing spondylitis can increase the risk of bladder cancer.^25^, ^72^ In other words, although knee OA and hip OA are not associated with bladder cancer, other types of OA may increase the risk of bladder cancer. Therefore, the relationship between OA at specific sites and bladder cancer still needs further study. Finally, due to the small effect size and number of SNPs in this MR study, especially the shortcomings in the methodology of MR analysis itself, we suggest that the causal effects of this MR method on any site OA and bladder cancer must be carefully estimated. The interpretation of this causal association emphasizes the importance of carefully considering the expected effects of intervention risk factors in a clinical setting, which requires further study.

In conclusion, there was a positive causal association between any site of OA and bladder cancer risk, but no major causal effect was found in site‐specific OA, such as knee OA and hip OA. This MR study conducted a detailed and complete analysis of the causative association between OA and bladder cancer risk, providing new cautiously declared evidence for early bladder cancer screening and prevention strategies in patients with OA.

## AUTHOR CONTRIBUTIONS

**Xi Zhang:** Methodology (equal); project administration (equal); resources (equal); visualization (equal); writing – original draft (equal). **Zengjin Wen:** Resources (equal); supervision (equal); writing – original draft (equal); writing – review and editing (equal). **Zixuan Xing:** Project administration (equal); supervision (equal); visualization (equal); writing – review and editing (equal). **Xiaoyu Zhou:** Methodology (equal); supervision (equal); writing – review and editing (equal). **Zhiluo Yang:** Methodology (equal); resources (equal); supervision (equal). **Ruijun Dong:** Methodology (equal); resources (equal); supervision (equal). **Jiao Yang:** Funding acquisition (equal); project administration (equal); supervision (equal); writing – review and editing (equal).

## FUNDING INFORMATION

Our work was funded by the National Natural Science Foundation of China (grant no. 82002794).

## CONFLICT OF INTEREST STATEMENT

The authors declare no conflict of interest.

## ETHICS STATEMENT

In this MR study, we used publicly available aggregate data; therefore, no separate ethical approval is required.

## Supporting information


Data S1.

## Data Availability

In this MR study, we used publicly available aggregate data; Therefore, no separate ethical approval is required and all the data is availability.
