# Supplementary material for: The causal relationship between osteoarthritis and bladder cancer: A Mendelian randomization study
Source: Cancer Med. 2023 Dec 15;13(1):e6829. doi: 10.1002/cam4.6829 (PMC10807596; doi:10.1002/cam4.6829)
Supplement: Supplementary file 1 — Data S1. [file CAM4-13-e6829-s001.docx]

Supplementary Table S1 any OA SNPs from the GWAS used as candidate genetic instruments in the Mendelian randomization analysis

| SNP | EA | OA | EAF | BETA | P-val. exposure | P-val. outcome | R^2 a^ | F-statistic ^b^ |
| --- | --- | --- | --- | --- | --- | --- | --- | --- |
| rs10042496 | C | T | 0.119 | 0.588 | 3.85E-06 | 0.528 | 0.073 | 35733.958 |
| rs150519705 | A | G | 0.009 | 2.330 | 1.74E-06 | 0.155 | 0.101 | 51140.174 |
| rs4328897 | C | A | 0.468 | 0.371 | 1.69E-06 | 0.444 | 0.068 | 33508.770 |
| rs647917 | C | T | 0.322 | 0.396 | 2.83E-06 | 0.254 | 0.069 | 33566.840 |
| rs77491146 | C | T | 0.078 | 0.701 | 3.17E-06 | 0.179 | 0.071 | 34797.223 |
| rs79756454 | C | T | 0.033 | 1.188 | 7.54E-07 | 0.343 | 0.090 | 45180.189 |
| rs9287903 | C | T | 0.262 | 0.458 | 2.51E-07 | 0.495 | 0.081 | 40205.522 |

EA: effect allele; OA: other allele; EAF: effect allele frequency of exposure of any OA; P-val: P-value for the genetic association.

^a^ R^2^ were calculated using the formula: 2×(1-EAF)×EAF×beta^2^, where beta is the estimated effect on any OA.

^b^ F-statistic were calculated using the formula: R^2^(N-2)/(1-R^2^), where N is the sample size of the GWAS for the any OA association.

Supplementary Table S2 knee OA SNPs from the GWAS used as candidate genetic instruments in the Mendelian randomization analysis

| SNP | EA | OA | EAF | BETA | P-val. exposure | P-val. outcome | R^2 a^ | F-statistic ^b^ |
| --- | --- | --- | --- | --- | --- | --- | --- | --- |
| rs111623565 | A | G | 0.004 | 1.309 | 2.76E-07 | 0.476 | 0.015 | 7058.973 |
| rs111956618 | C | A | 0.126 | -0.229 | 6.44E-07 | 0.882 | 0.012 | 5344.244 |
| rs11655443 | C | T | 0.031 | 0.409 | 4.71E-06 | 0.020 | 0.010 | 4619.657 |
| rs12792833 | T | G | 0.116 | 0.217 | 4.48E-06 | 0.076 | 0.010 | 4468.977 |
| rs140144990 | G | T | 0.011 | 0.687 | 4.48E-06 | 0.560 | 0.011 | 4924.709 |
| rs143339839 | G | A | 0.039 | 0.410 | 2.59E-07 | 0.720 | 0.013 | 5808.737 |
| rs148504141 | C | G | 0.009 | 0.776 | 4.78E-06 | 0.159 | 0.011 | 5080.665 |
| rs16944492 | C | T | 0.039 | 0.394 | 9.65E-07 | 0.471 | 0.012 | 5340.260 |
| rs4696079 | A | C | 0.463 | -0.145 | 1.72E-06 | 0.456 | 0.010 | 4805.810 |
| rs55642448 | T | C | 0.387 | 0.143 | 3.64E-06 | 0.705 | 0.010 | 4489.631 |
| rs56103030 | A | G | 0.018 | 0.563 | 2.04E-06 | 0.276 | 0.011 | 5199.072 |
| rs71604079 | A | G | 0.016 | 0.574 | 3.77E-06 | 0.747 | 0.011 | 4870.725 |
| rs740046 | G | A | 0.366 | -0.160 | 6.17E-07 | 0.021 | 0.012 | 5458.902 |
| rs7784284 | A | G | 0.153 | 0.194 | 3.75E-06 | 0.135 | 0.010 | 4504.344 |

EA: effect allele; OA: other allele; EAF: effect allele frequency of exposure of knee OA; P-val: P-value for the genetic association.

^a^ R^2^ were calculated using the formula: 2×(1-EAF)×EAF×beta^2^, where beta is the estimated effect on knee OA.

^b^ F-statistic were calculated using the formula: R^2^(N-2)/(1-R^2^), where N is the sample size of the GWAS for the knee OA association.

Supplementary Table S3 hip OA SNPs from the GWAS used as candidate genetic instruments in the Mendelian randomization analysis

| SNP | EA | OA | EAF | BETA | P-val. exposure | P-val. outcome | R^2 a^ | F-statistic ^b^ |
| --- | --- | --- | --- | --- | --- | --- | --- | --- |
| rs17610181 | A | G | 0.150 | 0.099 | 6.59E-10 | 0.984 | 0.002 | 958.264 |
| rs1800562 | A | G | 0.077 | 0.115 | 6.04E-08 | 0.908 | 0.002 | 725.274 |

EA: effect allele; OA: other allele; EAF: effect allele frequency of exposure of hip OA; P-val: P-value for the genetic association.

^a^ R^2^ were calculated using the formula: 2×(1-EAF)×EAF×beta^2^, where beta is the estimated effect on hip OA.

^b^ F-statistic were calculated using the formula: R^2^(N-2)/(1-R^2^), where N is the sample size of the GWAS for the hip OA association.

Supplementary Table S4 Association of SNPs used as candidate genetic instruments for any OA with confounders or bladder cancer

| SNP | Chr | Pos(hg38) | Trait | Excluded from MR analysis |
| --- | --- | --- | --- | --- |
| rs10042496 | 5 | 180423816 | None | No |
| rs150519705 | 14 | 34956419 | None | No |
| rs4328897 | 4 | 154093204 | None | No |
| rs647917 | 2 | 8412198 | None | No |
| rs77491146 | 3 | 65414440 | None | No |
| rs79756454 | 10 | 2245630 | None | No |
| rs9287903 | 2 | 168823612 | None | No |

Supplementary Table S5 Association of SNPs used as candidate genetic instruments for knee OA with confounders or bladder cancer

| SNP | Chr | Pos(hg38) | Trait | Excluded from MR analysis |
| --- | --- | --- | --- | --- |
| rs111623565 | 11 | 10784948 | Treatment with oxycodone hydrochloride | Yes |
| rs111956618 | 12 | 94985715 | None | No |
| rs11655443 | 17 | 32692008 | Self-reported giant cell or temporal arteritis | Yes |
| rs12792833 | 11 | 58777845 | None | No |
| rs140144990 | 3 | 9610618 | None | No |
| rs143339839 | 2 | 33221366 | None | No |
| rs148504141 | 12 | 51365679 | None | No |
| rs16944492 | 18 | 435867 | None | No |
| rs4696079 | 4 | 151342678 | None | No |
| rs55642448 | 8 | 10607245 | None | No |
| rs56103030 | 10 | 29311938 | None | No |
| rs71604079 | 4 | 37577483 | None | No |
| rs740046 | 7 | 31480533 | None | No |
| rs7784284 | 7 | 127515134 | None | No |

Supplementary Table S6 Association of SNPs used as candidate genetic instruments for hip OA with confounders or bladder cancer

| SNP | Chr | Pos（hg38） | Trait | Excluded from MR analysis |
| --- | --- | --- | --- | --- |
| rs17610181 | 17 | 61590592 | None | No |
| rs1800562 | 6 | 26092913 | Total cholesterol | Yes |
